# Myofibre Hypertrophy in the Absence of Changes to Satellite Cell Content Following Concurrent Exercise Training in Young Healthy Men

**DOI:** 10.3389/fphys.2021.625044

**Published:** 2021-06-04

**Authors:** Baubak Shamim, Donny M. Camera, Jamie Whitfield

**Affiliations:** Exercise and Nutrition Research Programme, Mary MacKillop Institute for Health Research, Australian Catholic University, Melbourne, VIC, Australia

**Keywords:** concurrent exercise, resistance exercise, endurance exercise, skeletal muscle, satellite cells

## Abstract

Concurrent exercise training has been suggested to create an ‘interference effect,’ attenuating resistance training-based skeletal muscle adaptations, including myofibre hypertrophy. Satellite cells support myofibre hypertrophy and are influenced by exercise mode. To determine whether satellite cells contribute to the ‘interference effect’ changes in satellite cell and myonuclear content were assessed following a period of training in 32 recreationally active males (age: 25 ± 5 year; body mass index: 24 ± 3 kg⋅m^–2^; mean ± SD) who undertook 12-week of either isolated (3 d⋅w^–1^) resistance (RES; *n* = 10), endurance (END; *n* = 10), or alternate day (6 d⋅w^–1^) concurrent (CET, *n* = 12) training. Skeletal muscle biopsies were obtained pre-intervention and after 2, 8, and 12 weeks of training to determine fibre type-specific cross-sectional area (CSA), satellite cell content (Pax7^+^DAPI^+^), and myonuclei (DAPI^+^) using immunofluorescence microscopy. After 12 weeks, myofibre CSA increased in all training conditions in type II (*P* = 0.0149) and mixed fibres (*P* = 0.0102), with no difference between conditions. Satellite cell content remained unchanged after training in both type I and type II fibres. Significant correlations were observed between increases in fibre type-specific myonuclear content and CSA of Type I (*r* = 0.63, *P* < 0.0001), Type II (*r* = 0.69, *P* < 0.0001), and mixed fibres (*r* = 0.72, *P* < 0.0001). Resistance, endurance, and concurrent training induce similar myofibre hypertrophy in the absence of satellite cell and myonuclear pool expansion. These findings suggest that myonuclear accretion via satellite cell fusion is positively correlated with hypertrophy after 12 weeks of concurrent training, and that individuals with more myonuclear content displayed greater myofibre hypertrophy.

## Introduction

Combining resistance- and endurance-based exercise training, or ‘concurrent exercise training,’ has previously been shown to impair strength and power adaptations compared to resistance training undertaken in isolation (Hickson, 1980; Craig et al., 1991; Hennessy and Watson, 1994; Kraemer et al., 1995; Dolezal and Potteiger, 1998; Bell et al., 2000; Häkkinen et al., 2003; Mikkola et al., 2012; Fyfe et al., 2016, 2018) and is referred to as the ‘interference effect.’ Notably, the result of concurrent exercise training on ‘interferences’ to lean mass gains relative to resistance training alone appear equivocal, with some studies showing greater gains in lean mass compared to resistance training alone (Kraemer et al., 1995; Bell et al., 2000; Rønnestad et al., 2012; Lundberg et al., 2013, 2014; Tomiya et al., 2017; Fyfe et al., 2018), while others have observed comparable (de Souza et al., 2013) or smaller gains in lean mass compared to resistance training alone (Sale et al., 1990; Timmons et al., 2018; Spiliopoulou et al., 2019). As such, understanding the ability of skeletal muscle to simultaneously adapt to divergent training stimuli is a topic that has received considerable attention (Nader, 2006; Wilson et al., 2012; Hamilton and Philp, 2013; Baar, 2014; Fyfe et al., 2014; Perez-Schindler et al., 2015; Murach and Bagley, 2016; Varela-Sanz et al., 2016; Coffey and Hawley, 2017; Doma et al., 2017; Berryman et al., 2018; Eddens et al., 2018; Fyfe and Loenneke, 2018; Hughes et al., 2018). Though the underlying cause of discrepancies in the degree of muscle hypertrophy achieved with concurrent versus resistance training remains unclear, it has recently been proposed that the potential for myofibre hypertrophy in response to chronic concurrent exercise training may be limited by satellite cell content (Babcock et al., 2012).

Satellite cells are myogenic precursor cells that reside between the sarcolemma and basal lamina (Mauro, 1961). In adult skeletal muscle, satellite cells exist in a quiescent state and are activated in response to various stimuli, such as exercise-induced mechanical stress, growth factors, and nutrients (Shamim et al., 2018b). Once activated, satellite cells can differentiate to form new myonuclei and increase transcriptional capacity, or return to quiescence to replenish the satellite cell pool through self-renewal (Kadi et al., 2005). Both satellite cell (Petrella et al., 2008; Verdijk et al., 2014; Moore et al., 2018) and myonuclear (Petrella et al., 2006, 2008) content have been shown to positively correlate with changes in myofibre cross-sectional area, suggesting an important relationship between myonuclear accretion and myofibre hypertrophy.

The degree of satellite cell activation and proliferation appears to be influenced by the mode of exercise performed. While evidence of satellite cell proliferation following resistance exercise is well documented (Crameri et al., 2004, 2007; Babcock et al., 2012; Farup et al., 2014; Snijders et al., 2014; Nederveen et al., 2015, 2017; Reidy et al., 2017a; Damas et al., 2018; Pugh et al., 2018), the capacity for acute endurance exercise to expand the satellite cell pool is less apparent (Snijders et al., 2011; Babcock et al., 2012; Nederveen et al., 2015), and may be dependent on type of exercise performed (Mackey et al., 2007) or intensity (Nederveen et al., 2015; McKenzie et al., 2016). Nonetheless, increases in satellite cell content have been reported following prolonged endurance training both in the presence (Charifi et al., 2003; Verney et al., 2008; Murach et al., 2016) and absence (Joanisse et al., 2013) of myofibre hypertrophy. In contrast, satellite cell activation and proliferation in response to concurrent exercise remains poorly understood.

To date, only two studies have evaluated changes in satellite cell content in response to concurrent exercise compared to isolated resistance exercise, with mixed results. Following a single bout of unilateral concurrent exercise incorporating moderate intensity cycling, Babcock et al. (2012) demonstrated that satellite cell proliferation was impaired in young, healthy males compared to resistance exercise performed alone in the contralateral leg. Based on this observation, the authors hypothesised that concurrent exercise impairs satellite cell responses and may contribute to limitations in myofibre hypertrophy observed with chronic concurrent exercise training. However, baseline satellite cell content prior to exercise was elevated in the leg that performed concurrent exercise compared to the one that performed resistance exercise only. Thus, the stable satellite cell content in response to concurrent exercise may have been indicative of a reduced need for proliferation rather than an inhibition of satellite cell activation *per se*. Recently, Pugh et al. (2018) demonstrated that a single bout of resistance or concurrent exercise incorporating high-intensity interval cycling results in comparable increases in satellite cell content in sedentary, overweight and obese middle-aged individuals, suggesting that concurrent exercise does not prevent satellite cell expansion if a high intensity endurance exercise stimulus is provided. While exercise order was the same in both studies (i.e., resistance exercise followed by endurance exercise), differences in the intensity of endurance exercise make direct comparisons between investigations cumbersome (Babcock et al., 2012; Pugh et al., 2018). Despite disparities in exercise protocols, to date, no studies have directly investigated the effect of chronic concurrent exercise training on changes in satellite cell content compared to isolated resistance or endurance training.

We have previously demonstrated that 12 weeks of concurrent exercise training resulted in similar lean mass gains as isolated resistance and endurance training, despite performing a greater volume of work (Shamim et al., 2018a). As satellite cell content appears to be associated with changes in myofibre cross-sectional area (Petrella et al., 2008; Verdijk et al., 2014; Moore et al., 2018), the aim of the present investigation was to evaluate whether changes in fibre type-specific satellite cell abundance are associated with the magnitude of hypertrophy achieved during concurrent training. Given the findings that performing a greater volume of work during acute concurrent exercise does not augment satellite cell proliferation compared to resistance or endurance exercise alone (Babcock et al., 2012; Pugh et al., 2018), it was hypothesised that chronic exercise training would result in similar increases in satellite cell content across all groups.

## Materials and Methods

### Experimental Overview

Using a parallel-groups design, participants were stratified according to lean body mass and allocated to either a resistance only (RES; *n* = 10), endurance only (END; *n* = 10) or concurrent resistance and endurance exercise training (CET; *n* = 12) group for 12 weeks. Consistent with previous recommendations to potentiate hypertrophy with concurrent training (Murach and Bagley, 2016), participants were given longer recovery periods (i.e., 24 h) between exercise sessions, cycling rather than running was selected for endurance exercise, and this was limited to ≤3 days per week. Measures of maximal strength, aerobic capacity, and anaerobic power, as well as body composition were performed pre- and post-intervention and have been previously reported (Shamim et al., 2018a; Timmins et al., 2020). Resting skeletal muscle biopsies were taken from the *vastus lateralis* at baseline (pre-intervention), and after 2, 8, and 12 weeks of training. The study was approved by the Australian Catholic University Human Research Ethics Committee and was carried out in accordance with the latest revision of the Declaration of Helsinki. This trial was registered with the Australian New Zealand Clinical Trials Registry (ACTRN12617001229369).

### Participants

Thirty-two young, healthy, and recreationally active males (age: 25 ± 5 year, body mass index: 24 ± 3 kg⋅m^–2^; mean ± SD) who had not participated in a structured exercise programme for ≥6 months preceding the study volunteered to participate. Participants were deemed healthy and eligible to participate based on their responses to a cardiovascular risk-factor questionnaire. All experimental procedures and risks associated with the study were explained to participants prior to providing written informed consent.

### Exercise Training

A detailed outline of the training programmes has been reported elsewhere (Shamim et al., 2018a). Briefly, for the duration of the intervention, participants in the RES and END group performed three non-consecutive days of training each week. Participants in the CET group trained 6 d⋅wk^–1^ and performed identical resistance and endurance programmes on alternating days as those in the RES and END groups, respectively. All training sessions were performed under the supervision of a member of the research team. Resistance training consisted of whole-body exercises with a focus on the leg press, knee extension and bench press movements, with these exercises performed at an intensity of ∼60–98% of 1RM. For example, leg press was progressed from participants doing 5 sets of 10 to 15 repetitions at 70% of 1RM in the first week of training to 2 sets of 2 repetitions at 97.5% of 1RM in the final week. If the participant was unable to achieve the prescribed number of repetitions, the weight was lowered by ∼5–10% for the following set to uphold the repetition scheme. Endurance cycle training was performed on Lode cycle ergometers and consisted of a mixture of a hill simulation ride of varying intensity (25–110% of maximum aerobic power (MAP), moderate-intensity continuous training at 50% MAP, moderate-intensity interval training at 70% MAP, and high-intensity interval training at 100% MAP. Moderate-intensity intervals were separated by a 60 s recovery period at ∼40% MAP, to establish a 2.5:1 or 5:1 work-to-rest ratio. High-intensity intervals were separated by 20–60 s recovery periods, completed at ∼40% MAP, to establish a 1:5, 1:2, or 1:1 work-to-rest ratio. All sessions were preceded by a standardised warm up for the respective training modality. Progressive overload was applied by periodically manipulating the number of sets and repetitions (resistance training), number of intervals (endurance training), and relative intensity of load the 12-week programme. All training programmes have been previously described in full detail and published elsewhere (Shamim et al., 2018a).

### Diet

A free-living, high-protein (2 g⋅kg^–1^⋅d^–1^) eating plan was implemented over the 12-week intervention. Participants completed daily food records and attended consultations with an Accredited Practising Dietitian on a fortnightly basis to monitor protein and energy intakes. All participants consumed ∼34 g of whey protein upon cessation of every training session to promote muscle protein synthesis (Macnaughton et al., 2016). Diet records were analysed for energy (kJ⋅kg^–1^), protein, carbohydrate, and fat (g⋅kg^–1^ for all macronutrients) to provide a daily average for the entire 12-week intervention. Daily averages of these dietary parameters have been previously published (Shamim et al., 2018a).

### Skeletal Muscle Biopsy

**FIGURE 1:**
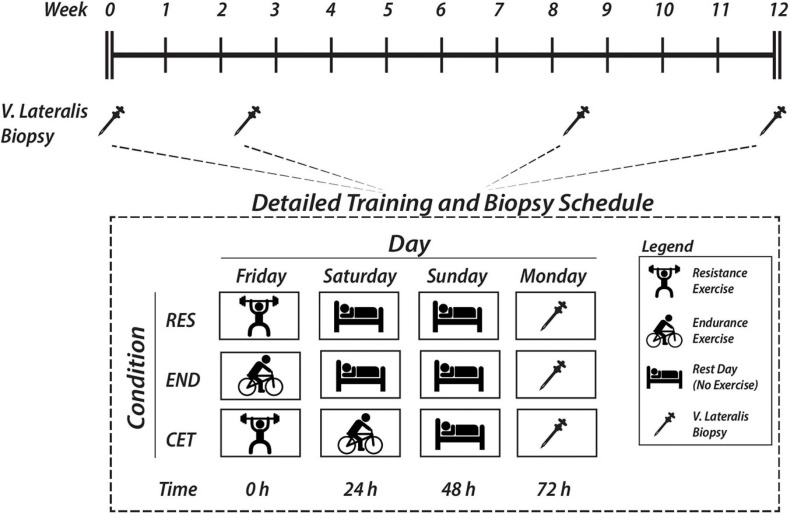
Schematic overview of study timeline and detailed vastus lateralis muscle biopsy sampling times following endurance (END), resistance (RES), or concurrent (CET) training.

Resting skeletal muscle biopsies were collected after an overnight fast pre-intervention and 72 h after the last exercise session of weeks 2, 8, and 12 from a distal portion of the *vastus lateralis* using a Bergstrom needle modified for manual suction under local anaesthesia (2% Xylocaine). Each muscle biopsy was obtained from a separate site 2–3 cm proximal to the previous biopsy site on the same leg. Biopsies in the CET condition were taken 72 h after resistance exercise to determine the effects of endurance exercise performed on alternate days on satellite cell expansion (Figure 1). Samples were immediately frozen in liquid nitrogen or embedded in optimum cutting temperature compound (Scigen) and frozen in liquid nitrogen-cooled isopentane. Samples were stored at −80^*o*^C for subsequent analysis.

### Immunohistochemistry

Muscle cross-sections (7 μm) were stained for cross-sectional area (CSA), fibre type, myonuclei, and satellite cells. Cross-sections were fixed in 2% formaldehyde, washed, blocked for 90 min (1X phosphate-buffered saline (PBS) containing 2% bovine serum albumin (BSA), 5% fetal bovine serum (FBS), 0.2% Triton X-100, 2% goat serum (GS), and 0.02% NaN_3_), and then incubated in primary antibodies for Pax7 (1:4, DSHB) and laminin (1:250, ab11575, Abcam) overnight at 4^*o*^C. Sections were washed, then incubated for 2 h in secondary antibodies for Alexa Fluor 488 (#A32731) and 594 (#A11032; Invitrogen). Sections were re-fixed, washed, blocked for 2 h (5% GS containing 0.01% Triton x-100 and 0.05% NaN_3_), and then incubated in primary antibody for MHCI (1:4, A.4.951, DSHB) overnight at 4^*o*^C. Sections were washed then incubated for 2 h in secondary antibody (Alexa Fluor 488, #A11029, Invitrogen), nuclei were labelled with 4’,6-diamidino-2-phenylindole (DAPI, 1:20,000, Life Technologies), and cover slips affixed with ProLong^TM^ Diamond Antifade Mountant (Invitrogen).

Staining for fibre CSA and fibre-typing was undertaken following fixation by blocking sections for 2 h (5% GS containing 0.01% Triton X-100 and 0.05% NaN_3_), then incubating in primary antibodies for MHCI and laminin overnight at 4^*o*^C. Sections were washed, incubated in appropriate secondary antibodies, washed again, and then affixed with cover slips. As an antibody for MHCIIa was not used, fibres that were negative for MHCI are referred to as MHCII, which includes MHCIIa and MHCIIx fibre types (Babcock et al., 2012).

All antibodies were diluted in 1% BSA, and secondary antibodies diluted 1:500. Images were observed under an EVOS^TM^ FL Auto 2 microscope (Invitrogen) at 20X (nuclei) and 10X (CSA) objectives. An average of 218 ± 30 (90 ± 31 Type I, 128 ± 35 Type II) and 230 ± 44 (98 ± 24 Type I, 132 ± 38 Type II) fibres were assessed per participant at each time point for CSA and satellite cell enumeration, respectively. Peripheral muscle fibres that displayed irregular edge staining patterns or disrupted cell membranes and longitudinal fibres (circularity < 0.6) were excluded from analyses. Images were analysed using ImageJ-Fiji.

### Statistical Analysis

The present study is a secondary analysis from previously published work powered to detect a medium effect (Cohen’s *f* = 0.25) with a significance level of α = 0.05 and 80% power for change in lean body mass (Shamim et al., 2018a). Thus, outcome measures including change in fibre CSA, satellite cell content, and myonuclear content are exploratory. Linear mixed-effect (LME) model, fit by restricted maximum likelihood estimate with random intercept for subject was used to test the effect of training condition on myofibre CSA, myofibre-type distribution, myonuclear number, and satellite cell content. Interactions for training condition × time were tested by the same LME. Where LME revealed significance, a Bonferroni *post hoc* test for pair-wise comparisons was performed. The relationships between baseline satellite cell content and change in fibre CSA as well as change in myonuclear content and change in fibre CSA were determined by calculating Pearson correlation coefficients (*r*). Statistical significance was set at *P* < 0.05. Data are presented as mean ± standard deviation. Statistical analysis was performed using R (v3.5.2).

## Results

### Fibre CSA and Distribution

**FIGURE 2:**
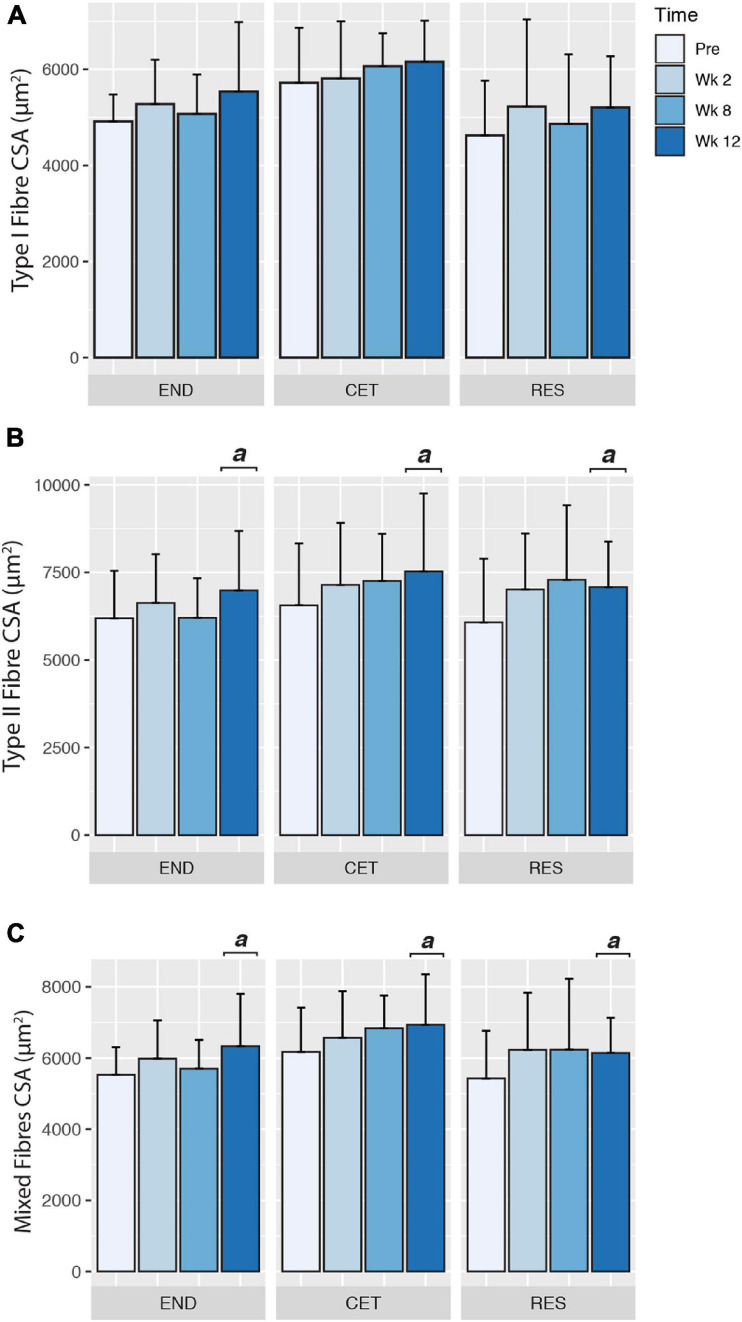
Changes to myosin heavy chain type I **(A)**, myosin heavy chain type II **(B)**, and mixed **(C)** myofibre cross-sectional area (CSA) in response to endurance (END; *n* = 10), resistance (RES; *n* = 10), or concurrent (CET; *n* = 12) training. a, significantly different from Pre time point (*P* < 0.05). Values are presented as Mean ± SD.

**FIGURE 3:**
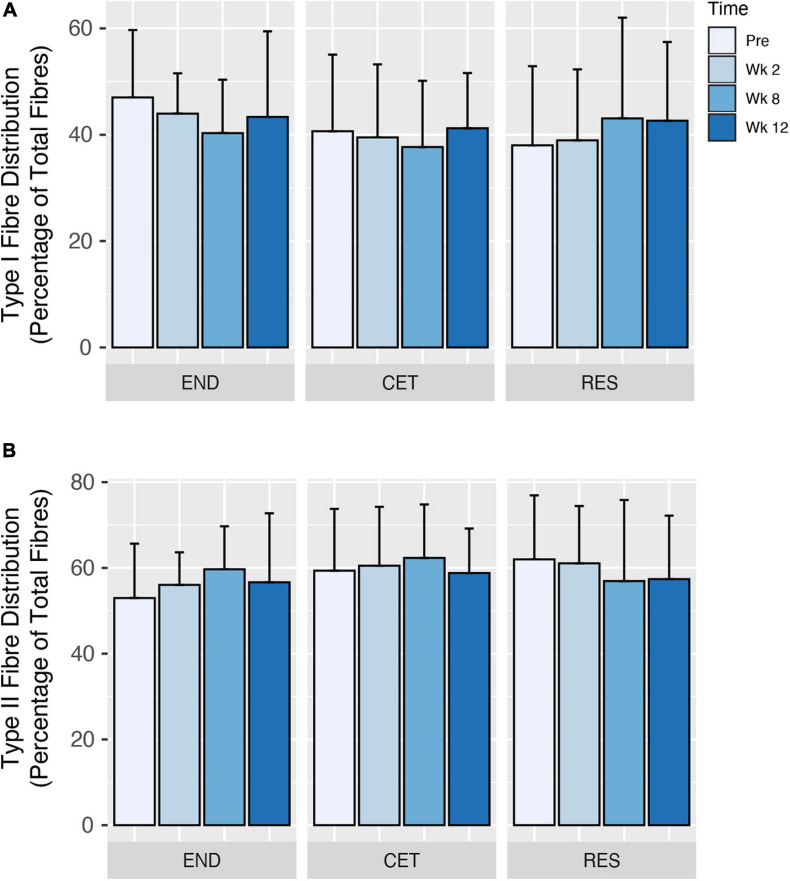
Changes to type I **(A)** and type II **(B)** fibre distribution in response to endurance (END; *n* = 10), resistance (RES; *n* = 10), or concurrent (CET; *n* = 12) training. Values are presented as Mean ± SD.

Following 12 weeks of training, there was a main effect of condition (*P* = 0.0317), but not time or condition by time for an increase in Type I fibre CSA. However, when corrected for multiple comparisons, *post hoc* analysis revealed no significant differences in Type I fibre CSA for condition (Figure 2A). Despite employing different training modalities, only a main effect of time (*P* = 0.0474), but not condition or condition by time was observed for an increase in Type II fibre CSA. *Post hoc* analysis revealed an increase in Type II fibre CSA at week 12 compared to Pre (*P* = 0.0149; Figure 2B). Similarly, when the mean CSA of both Type I and II fibres was assessed, there was a main effect of time (*P* = 0.0487), but no main effect for condition or condition by time. *Post hoc* analysis revealed an increase in mixed fibres CSA at week 12 compared to Pre (*P* = 0.0102; Figure 2C). Fibre-type distribution was unaffected by the training intervention, and displayed no main effect of condition, time or condition by time (Figures 3A,B).

### Satellite Cell Content

**FIGURE 4:**
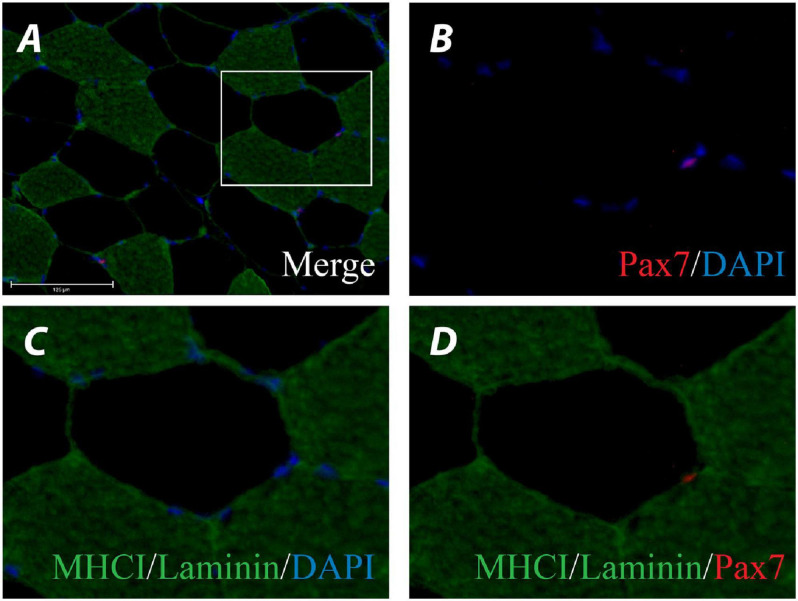
Representative image of a myosin heavy chain (MHC) I/Laminin/Pax7/DAPI stain of a muscle cross-section **(A)**. Channel view of Pax7/DAPI **(B)**, MHCI/Laminin/DAPI **(C)**, and MHCI/Laminin/Pax7 **(D)**.

**FIGURE 5:**
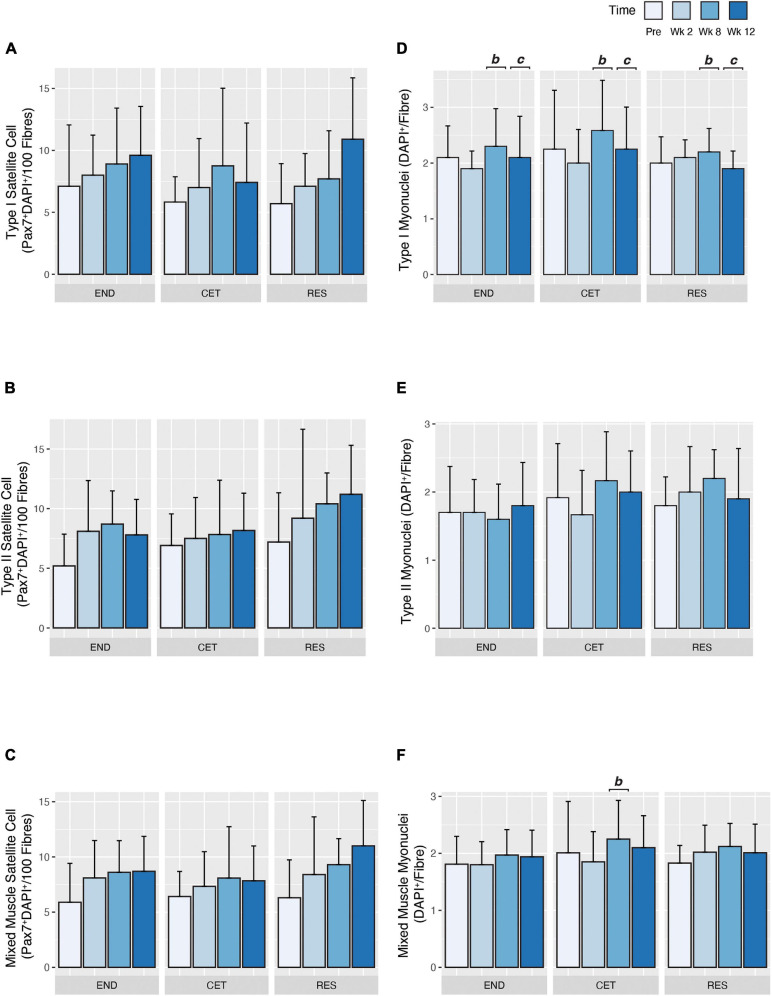
Fibre type-specific satellite cell **(A–C)** and myonuclear **(D–F)** expansion in response to endurance (END, *n* = 10), resistance (RES; *n* = 10), or concurrent (CET; *n* = 12) training. Change in myosin heavy chain type I **(A)**, myosin heavy chain type II **(B)**, and mixed **(C)** myofibre satellite cell content. Change in myosin heavy chain type I **(D)**, myosin heavy chain type II **(E)**, and mixed **(F)** myofibre myonuclear content. b, significantly different from week 2 time point (*P* < 0.05); c, significantly different from week 8 time point (*P* < 0.05). Values are presented as Mean ± SD.

Satellite cells were determined as staining positive for both DAPI and Pax7 and having a location between the basal lamina and the plasma membrane of myofibres (see Figure 4 for representative stain). In response to 12 weeks of exercise training there was no main effect of condition, time or condition by time for change in Type I satellite cell content (Figure 5A). Similarly, there was no main effect of condition, time or condition by time for change in Type II satellite cell content (Figure 5B). There was no main effect of condition, time or condition by time for change in mixed fibre-type satellite cell content (Figure 5C).

### Myonuclear Content and Domain

In response to training, there was a main effect of time (*P* = 0.044), but not condition or condition by time for an increase in Type I myonuclear content. *Post hoc* analysis revealed that myonuclear content was greater at week 8 compared to week 2 f (*P* = 0.0031) and week 12 (*P* = 0.0410; Figure 5D). Conversely, training did not alter Type II myonuclear content as no main effect of condition, time or condition by time was observed (Figure 5E). When the mean myonuclear content of both Type I and II fibres was assessed, a main effect of time (*P* = 0.0302), and condition by time (*P* = 0.0350), but not condition was observed. *Post hoc* analysis revealed mixed fibre myonuclear content was greater at week 8 (2.25 ± 0.679 DAPI^+^/Fibre) compared to week 2 (1.85 ± 0.532 DAPI^+^/Fibre; *P* = 0.0297; Figure 5F).

Despite observing an increase in Type I myonuclear content, no main effect of condition, time or condition by time was seen for Type I myonuclear domain. Conversely, Type II myonuclear domain increased in response to training as a main effect of time (*P* = 0.0341), but not condition or condition by time. However, when corrected for multiple comparisons, *post hoc* analysis revealed no significant differences in Type II myonuclear domain with time. Likewise, when the mean myonuclear domain of both Type I and II fibres was assessed, no main effect of condition, time or condition by time was present (data not shown).

### Pearson’s Correlation Coefficients of Muscle Characteristics

**FIGURE 6:**
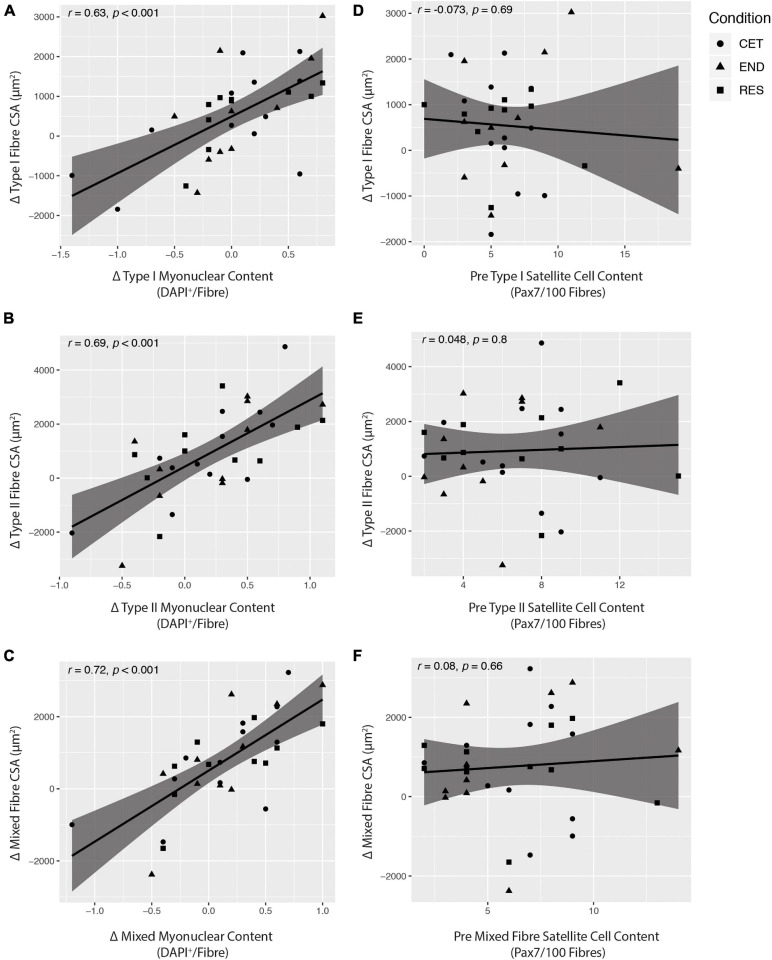
Pearson correlation coefficients (r) showing the relationship between fibre type-specific changes in cross-sectional area (CSA) and myonuclear content **(A–C)** and fibre type-specific changes in CSA and pre-intervention baseline satellite cell content **(D–F)**. Statistical significance was set at *P* < 0.05. CET, concurrent training; END, endurance training; RES, resistance training.

There were significant correlations between increases in fibre type-specific myonuclear content and increases in fibre CSA of Type I (*r* = 0.63, *P* < 0.0001; Figure 6A), Type II (*r* = 0.69, *P* < 0.0001; Figure 6B), and mixed fibres (*r* = 0.72, *P* < 0.0001; Figure 6C). Conversely, there was no relationship between pre-intervention fibre type-specific satellite cell content and increases in fibre CSA of Type I (*r* = −0.073, *P* = 0.69; Figure 6D), Type II (*r* = 0.048, *P* = 0.8; Figure 6E), or mixed fibres (*r* = −0.08, *P* = 0.66; Figure 6F). There was no relationship between change in fibre type-specific satellite cell content and increase in fibre CSA for Type I (*r* = 0.22, *P* = 0.23), Type II (*r* = 0.24, *P* = 0.19), or mixed fibres (*r* = 0.23, *P* = 0.22).

## Discussion

The aim of the present investigation was to evaluate fibre type-specific changes in satellite cell abundance are associated with the magnitude of hypertrophy achieved during concurrent training. The present study shows that in response to 12 weeks of endurance, resistance, and concurrent training, Type II and mixed fibre CSA and Type I myonuclear content are increased without a significant change in total satellite cell content, with no differences across training modalities. This is consistent with the changes in lean mass previously reported following the intervention, as the increase in lean mass was similar across CET, RES, and END, despite CET completing a greater overall training volume than RES and END (Shamim et al., 2018a). It has been suggested concurrent exercise training may augment the hypertrophic response to resistance exercise training in some circumstances as aerobic exercise training alone can induce hypertrophy (Murach and Bagley, 2016). However, despite implementing longer between-session recovery and limiting endurance exercise to three sessions per week in the form of cycling in line with recommendations to augment hypertrophy with concurrent training (Murach and Bagley, 2016), no differences in the magnitude of myofibre hypertrophy achieved were observed between modalities. Notably, previous reports of augmented hypertrophy (e.g., increased myofibre CSA) with concurrent training compared to resistance training alone have been observed during shorter (i.e., ≤7 weeks) interventions (Lundberg et al., 2013; Kazior et al., 2016). However, longer training interventions (i.e., ≥20 weeks), have failed to observe any differences in myofibre CSA hypertrophy compared to resistance training (Sale et al., 1990; Häkkinen et al., 2003). While the exact timeline of muscle hypertrophy remains ambiguous (Damas et al., 2015), it is possible that disparities in the magnitude of myofibre hypertrophy observed with concurrent training between previous work (Lundberg et al., 2013; Kazior et al., 2016) and the present study are due to differences in the length of the intervention, training variables (e.g., frequency, intensity, and duration), as well as training and nutritional status of the participants. Nevertheless, while the findings in the present study support the recent recommendations for concurrent exercise training prescription to promote hypertrophy (Murach and Bagley, 2016), they do not appear to facilitate an greater hypertrophic response compared to resistance or endurance training alone over 12 weeks at the myofibre CSA level.

It is unclear why the greater volume of work completed by participants in the concurrent training group did not induce a greater increase in myofibre CSA. It has been hypothesised that the potential for myofibre hypertrophy to occur with chronic concurrent training may be limited by satellite cells (Babcock et al., 2012). Evidence for impaired satellite cell responses with concurrent exercise compared to resistance exercise alone has been observed when a bout of moderate intensity resistance exercise is followed immediately by moderate intensity continuous cycling in young, healthy males (Babcock et al., 2012). However, performing moderate intensity resistance exercise followed immediately by high-intensity interval cycling results in an increase in Type I fibre satellite cell content comparable to resistance exercise alone in sedentary, overweight and obese, middle-aged individuals (Pugh et al., 2018). In the present study, both moderate intensity continuous and high intensity interval cycling was implemented, however, contrary to the initial hypothesis, no significant increase in satellite cell content was detected in response to any training modality.

Activation of fibre type-specific satellite cells has also been shown to be attenuated following concurrent exercise compared to resistance exercise (Pugh et al., 2018). As satellite cell activation can generate both progeny for self-renewal of the satellite cell pool and myogenic precursors to undergo terminal differentiation (Kuang et al., 2007), this blunted response may underlie the limited satellite cell expansion previously observed following concurrent exercise (Babcock et al., 2012; Pugh et al., 2018). Increases in satellite cell activation have been observed as soon as 9 h after a single bout of concurrent exercise in young, healthy males (Snijders et al., 2012), with subsequent increases in satellite cell content peaking ∼72 h post-exercise (Snijders et al., 2015). Given the transient nature of this response, it is possible that the absence of active satellite cells at 96 h after concurrent exercise previously reported (Babcock et al., 2012; Pugh et al., 2018) may be due to post-exercise biopsy timing. In the present study, muscle biopsies were collected 48–72 h post-exercise in order to capture peak increases in satellite cell content. Though satellite cell activation was not directly assessed in the present study, a stable number of satellite cells and increased number of myonuclei was observed after 8 weeks in Type I fibres in all training conditions. This finding suggests that satellite cell activation and proliferation occurred in order to maintain a stable satellite cell pool. Furthermore, training-induced increases in myonuclear content in response to concurrent training were not different from isolated endurance or resistance training and occurred without fibre-type specific hypertrophy. Accordingly, concurrent training does not prevent satellite cell differentiation and myonuclear accretion. Collectively, these observations suggest that chronic concurrent training does not inhibit satellite cell activation or myogenesis.

While differences in exercise intensity, training status of participants, and baseline satellite cell content may underlie disparities in satellite cell expansion previously observed with concurrent exercise (Babcock et al., 2012; Pugh et al., 2018), it is difficult to reconcile why no increase in satellite cell content was observed in response to either resistance or endurance training alone. Given the hypertrophy observed in Type II and mixed fibre CSA in the absence of myonuclear addition, it is possible that the existing myonuclei were able to support the degree of hypertrophy achieved and that a large magnitude of expansion to the satellite cell pool to promote myogenesis was not needed. It has been suggested that myonuclear content may regulate the capacity for myofibre hypertrophy. According to the myonuclear domain theory, a single myonucleus can only provide sufficient transcriptional capacity over a finite amount of cytoplasm (Cheek, 1985; Allen et al., 1999). During periods of extensive myofibre hypertrophy, increases in myofibre CSA are accompanied by an increase in cell volume, which results in a strain on the myonuclear domain (i.e., μm^2^ fibre area/myonucleus). In turn, myofibre hypertrophy can occur as a result of increasing the size of existing myonuclear domains or by increasing the absolute number of domains within the myofibre (Edgerton and Roy, 1991; Kadi and Thornell, 2000). Contrary to this idea, Kirby et al. (2016) demonstrated that existing myonuclei are able to increase transcriptional capacity to support hypertrophy in the absence of satellite cell-dependent myonuclear accretion. Thus, it is possible that the hypertrophy observed in Type II and mixed fibre CSA may be influenced by enhanced transcriptional responses of existing myonuclei.

It has been hypothesised that myonuclear addition only occurs when the myonuclear domain exceeds an absolute ‘ceiling’ (∼2,250 μm^2^) (Petrella et al., 2006, 2008), or myofibre hypertrophy exceeds a relative magnitude (∼26%) (Kadi et al., 2004). While the average Type II myonuclear domain (∼4,200 μm^2^) in the present study exceeds the aforementioned theoretical myonuclear domain ‘ceiling,’ this value is comparable with others previously reported (Karlsen et al., 2015). Additionally, despite a lack of hypertrophy, there was an increase in myonuclear number in Type I fibres observed in the present study, which has recently been demonstrated following 12 weeks of resistance training in older adults (Moro et al., 2020). Collectively, these findings do not support the rationale that hypertrophy must exceed a relative threshold to permit myonuclear addition. Likewise, the hypertrophy observed in Type II fibres occurred without a prior increase in myonuclear domain and demonstrates that changes in myonuclear domain size do not precede myofibre hypertrophy. These findings are supported by previous work demonstrating that myofibre hypertrophy in response to 12 weeks of resistance training occurs without prior changes in myonuclear domain size in young, healthy men (Snijders et al., 2016). In contrast, in the present study, the average Type II and mixed fibre-type hypertrophy observed was ∼15% and ∼13%, respectively, which occurred in the absence of myonuclear addition or significant expansion in the myonuclear domain. Taken in isolation, this observation would be in agreement with the notion that a ∼26% increase in CSA is needed to evoke myonuclear addition (Kadi et al., 2004). However, collectively the changes in myonuclear numeration and domain sizes across fibre-types are equivocal as it pertains to the threshold hypothesis. Indeed, the concept of a universal myonuclear threshold has recently been challenged (Conceição et al., 2018), which is consistent with the equivocal responses found in the current study across fibre-types. These observations therefore highlight that the myonuclear domain does not dictate increases to myonuclear content and does not appear to be a limiting factor in the degree of myofibre hypertrophy achieved with concurrent training.

While there is considerable debate around the notion that satellite cells are required to facilitate overload-induced myofibre hypertrophy (Petrella et al., 2006, 2008; Verdijk et al., 2009, 2014; Bellamy et al., 2014; Karlsen et al., 2015; Dirks et al., 2017; McCarthy et al., 2017; Murach et al., 2017; Reidy et al., 2017b), current evidence indicates that a positive correlation exists between satellite cell-mediated myonuclear accumulation and myofibre hypertrophy (Petrella et al., 2006, 2008; Verdijk et al., 2010, 2014; Bellamy et al., 2014; Reidy et al., 2017b). In accordance, higher baseline satellite cell content has been associated with a greater magnitude of myofibre hypertrophy achieved after a period of resistance training (Petrella et al., 2008). In the current study, there was no relationship between baseline satellite cell content and increases in Type I, Type II, or mixed myofibre CSA. However, a positive correlation was observed for increases in fibre type-specific myonuclear content and increases in myofibre CSA. These observations are consistent with previous works (Petrella et al., 2006, 2008; Verdijk et al., 2010, 2014; Bellamy et al., 2014; Reidy et al., 2017b) demonstrating that increases in myonuclear number are tightly coupled to increases in myofibre CSA. As myonuclear accretion is dependent on the fusogenic capacity of satellite cells (McCarthy et al., 2011; Fry et al., 2014), the present findings suggest that satellite cells directly support enhanced hypertrophy through myonuclear accretion following resistance, endurance, and concurrent exercise training.

Previous work has demonstrated that satellite cell proliferation is dependent on exercise mode (Mackey et al., 2007; Babcock et al., 2012; Nederveen et al., 2015) and contraction type (Farup et al., 2014), with resistance-type exercise contractions stimulating the greatest response (Snijders et al., 2015). It is therefore unclear why exercise modality did not influence satellite cell proliferation in the current study. One possible explanation is the finding that the relative intensity of exercise is also critical factor in provoking a satellite cell response (Nederveen et al., 2015; McKenzie et al., 2016). Given the recreationally active training status of the participants, it is possible that the training protocols were not strenuous enough to elicit a differential satellite cell response between training modalities. Recent evidence has shown that exercise promotes myogenesis in a load-dependent manner in mice (Masschelein et al., 2020), suggesting that higher loads performed during exercise may also illicit greater satellite cell responses in human skeletal muscle. Likewise, as resistance exercise was not performed to volitional muscle failure in the present study, the relative intensity of training may not have been sufficient to maximally stimulate muscle hypertrophy (Mitchell et al., 2012), but, instead, only produced modest hypertrophy comparable to endurance cycle training. While expansions in the satellite cell pool have been observed in the absence of hypertrophy (Joanisse et al., 2013), whether or not a greater degree of myofibre hypertrophy would have promoted an increased demand for satellite cell proliferation to support myogenesis remains unresolved. Thus, future studies evaluating relative load and exercise intensity (i.e., training to volitional muscle failure) of concurrent training are needed to fully appreciate the contribution of modulating training variables (such as load or intensity) on satellite cell responses.

We acknowledge there are several limitations in the present study. As the present study is a follow up analysis to a study investigating changes in lean body mass to concurrent exercise training (Shamim et al., 2018a) and the first to employ a chronic, parallel groups design incorporating resistance, endurance, and concurrent exercise training, it is possible that the current sample size may not be large enough to detect modest changes to sensitive variables, such as satellite cells. However, to the best of our knowledge, no chronic training study has utilised satellite cell enumeration as a primary outcome. It is therefore difficult to accurately calculate the minimum required sample size in order to be sufficiently powered to detect significantly meaningful changes in this outcome. Similarly, due to the relatively small sample size analysed in the current study, large variations in changes to fibre CSA may have masked the detection of significant differences in the magnitude of muscle hypertrophy achieved between training conditions. Next, biopsies were collected 48 h after the last bout of endurance exercise in the CET condition and 72 h after the last bout of exercise in RES and END. As the number of satellite cells has been shown to peak ∼72 h after exercise (Snijders et al., 2015), consideration must be given to discrepancies in biopsy sampling time between CET and END conditions. Thus, whether CET alters satellite cell and/or myonuclear content 72 h after endurance exercise in cannot be distinguished with the selected biopsy sampling time. Likewise, as no pre-exercise biopsies were collected during weeks 2, 8, or 12, it is not possible to determine whether training altered satellite cell activation. It has recently been shown that chronic resistance training enhances the activation of satellite cells in response to an acute bout of exercise (Nederveen et al., 2017). Thus, in the present study only static measures of satellite cell abundance were assessed, making it difficult to determine specific satellite cell responses to exercise. Future investigations assessing markers of satellite cell activation (i.e., MyoD, Myf5, and Myogenin) are required to understand if chronic concurrent training alters satellite cell activation in response to an acute exercise stimulus. Likewise, previous training history may alter satellite cell activation and myonuclear accretion in response to exercise stimuli (Nederveen et al., 2017). While myonuclear density does not appear to be sustained after periods of detraining (Dungan et al., 2019), it is possible that epigenetic modifications to within myonuclei may contribute to growth responses to future exercise training (Seaborne et al., 2018). Thus, future investigations examining long-term training history will be prudent for understanding mechanisms that mediate satellite cell-dependent hypertrophy.

## Conclusion

This is the first investigation to assess changes in satellite cell and myonuclear content following a period of chronic concurrent exercise training compared to isolated resistance and endurance training. The findings demonstrate that resistance, endurance, and concurrent training induce myofibre hypertrophy in the absence of significant expansion to the satellite cell, however, myonuclear accretion via satellite cell fusion is positively correlated with hypertrophy after 12 weeks of exercise training. Despite clear differences between training modalities with regards to physiological adaptations (i.e., one-repetition maximum strength, maximum anaerobic power, and VO2_*peak*_) (Shamim et al., 2018a), implementing the current recommended strategies to maximise hypertrophic potential with chronic concurrent training do not result in greater myofibre hypertrophy, satellite cell pool expansion, or myonuclear accretion compared to endurance or resistance training alone. Likewise, myonuclear domain size remains stable throughout chronic endurance, resistance, and concurrent training, and, as such, does not appear to be a critical mediator in myonuclear accretion or limit the degree of hypertrophy achieved with concurrent training. The current data suggest that changes in myonuclear content are not prerequisite to changes in myofibre hypertrophy but do appear to be associated with the magnitude of myofibre hypertrophy achieved in young, healthy males.

## Data Availability Statement

The raw data supporting the conclusions of this article will be made available by the authors, without undue reservation.

## Ethics Statement

The studies involving human participants were reviewed and approved the Australian Catholic University Human Research Ethics Committee (2016-54H). The patients/participants provided their written informed consent to participate in this study.

## Author Contributions

BS and DC conceptualised and designed the study. BS performed the experiments and analysed the data. BS and JW interpreted the results and drafted and edited the manuscript. All authors approved the final version of the manuscript.

## Conflict of Interest

The authors declare that the research was conducted in the absence of any commercial or financial relationships that could be construed as a potential conflict of interest.
