# Corrigendum: The Relationship Between Parental Attachment and Mobile Phone Dependence Among Chinese Rural Adolescents: The Role of Alexithymia and Mindfulness

**DOI:** 10.3389/fpsyg.2021.717059

**Published:** 2021-07-28

**Authors:** Xiaoqing Li, Chenrui Hao

**Affiliations:** ^1^College of Psychology and Sociology, Shenzhen University, Shenzhen, China; ^2^Shenzhen Key Laboratory of Affective and Social Cognitive Science, Shenzhen University, Shenzhen, China

**Keywords:** parental attachment, mobile phone dependence, alexithymia, mindfulness, adolescents

In the original article, we neglected to include the funding information. The corrected Funding statement is shown below:

This study was supported by the Department of Education in Guangdong Province of China, Grant No. 2016WQNCX130; Educational Science Research Institute of Shenzhen, Grant No. zdfz18007; and Shenzhen Social Science Association, Grant No. SZ2019D055 to XL.

The authors apologize for this error and state that this does not change the scientific conclusions of the article in any way. The original article has been updated.

## Publisher's Note

All claims expressed in this article are solely those of the authors and do not necessarily represent those of their affiliated organizations, or those of the publisher, the editors and the reviewers. Any product that may be evaluated in this article, or claim that may be made by its manufacturer, is not guaranteed or endorsed by the publisher.

