# Corrigendum: Myofibre Hypertrophy in the Absence of Changes to Satellite Cell Content Following Concurrent Exercise Training in Young Healthy Men

**DOI:** 10.3389/fphys.2021.736848

**Published:** 2021-07-28

**Authors:** Baubak Shamim, Donny M. Camera, Jamie Whitfield

**Affiliations:** Exercise and Nutrition Research Programme, Mary MacKillop Institute for Health Research, Australian Catholic University, Melbourne, VIC, Australia

**Keywords:** concurrent exercise, resistance exercise, endurance exercise, skeletal muscle, satellite cells

In the original article, there was an error. The introduction incorrectly stated that work by Lundberg et al., 2013, 2014, de Souza et al., 2013, and Kazior et al., 2016 demonstrated reduced lean mass gains in the concurrent (resistance plus endurance) exercise trained group compared to resistance alone. This has been amended below. These papers were discussed in the correct context throughout the rest of the manuscript.

A correction has been made to *Introduction, Paragraph 1*:

Combining resistance- and endurance-based exercise training, or ‘concurrent exercise training,’ has previously been shown to impair strength and power adaptations compared to resistance training undertaken in isolation (Hickson, 1980; Craig et al., 1991; Hennessy and Watson, 1994; Kraemer et al., 1995; Dolezal and Potteiger, 1998; Bell et al., 2000; Häkkinen et al., 2003; Mikkola et al., 2012; Fyfe et al., 2016, 2018) and is referred to as the ‘interference effect.’ Notably, the result of concurrent exercise training on ‘interferences’ to lean mass gains relative to resistance training alone appear equivocal, with some studies showing greater gains in lean mass compared to resistance training alone (Kraemer et al., 1995; Bell et al., 2000; Rønnestad et al., 2012; Lundberg et al., 2013, 2014; Tomiya et al., 2017; Fyfe et al., 2018), while others have observed comparable (de Souza et al., 2013) or smaller gains in lean mass compared to resistance training alone (Sale et al., 1990; Timmons et al., 2018; Spiliopoulou et al., 2019). As such, understanding the ability of skeletal muscle to simultaneously adapt to divergent training stimuli is a topic that has received considerable attention (Nader, 2006; Wilson et al., 2012; Hamilton and Philp, 2013; Baar, 2014; Fyfe et al., 2014; Perez-Schindler et al., 2015; Murach and Bagley, 2016; Varela-Sanz et al., 2016; Coffey and Hawley, 2017; Doma et al., 2017; Berryman et al., 2018; Eddens et al., 2018; Fyfe and Loenneke, 2018; Hughes et al., 2018). Though the underlying cause of discrepancies in the degree of muscle hypertrophy achieved with concurrent versus resistance training remains unclear, it has recently been proposed that the potential for myofibre hypertrophy in response to chronic concurrent exercise training may be limited by satellite cell content (Babcock et al., 2012).

The authors apologize for this error and state that this does not change the scientific conclusions of the article in any way. The original article has been updated.

## Publisher's Note

All claims expressed in this article are solely those of the authors and do not necessarily represent those of their affiliated organizations, or those of the publisher, the editors and the reviewers. Any product that may be evaluated in this article, or claim that may be made by its manufacturer, is not guaranteed or endorsed by the publisher.
